# The orphan histidine kinase TodK controls *Myxococcus xanthus* biofilm development by inactivating the CRP/Fnr homolog, MrpC

**DOI:** 10.64898/2026.07.28.741289

**Published:** 2026-07-29

**Authors:** Christopher Mataczynski, Maike Glaser, Stuart Huntley, Penelope I. Higgs

## Abstract

Environmental bacteria have abundant signaling systems wired into complex gene regulatory networks to adapt to fluctuating conditions. In *Myxococcus xanthus* , starvation triggers a developmental program (specialized biofilm) that produces spore-filled multicellular fruiting bodies surrounded by a distinct quiescent state termed peripheral rods. Fruiting body structure as well as the proportion of cells following each fate can be tuned by a large repertoire of signaling proteins, including numerous orphan histidine kinases. Here, we focus on the histidine kinase TodK which was previously demonstrated to influence developmental progression. We find that loss of TodK produces distinct developmental phenotypes that vary with environmental conditions. To quantify these effects, we developed an image-analysis pipeline that measures aggregation and fruiting body patterning during development on nutrient-limited agar. These analyses revealed the *todK* mutant precociously aggregates particularly at the peripheries of the colony. Under submerged-culture conditions, initial production of aggregates was not accelerated but aggregates exhibited accelerated progression to mature fruiting bodies. Overexpression of active TodK completely blocked fruiting body formation. Molecular analyses demonstrated that TodK overproduction suppressed expression of core developmental regulators including FruA and CsgA (C-signal). Interestingly, protein accumulation of MrpC, necessary for expression of both FruA and the C-signal was not significantly perturbed suggesting TodK silences MrpC transcriptional activity. Together, these findings establish TodK as a modulator of developmental progression and demonstrate how quantitative phenotyping approaches can reveal biologically meaningful functions for orphan histidine kinases whose mutant phenotypes might otherwise appear subtle.

**Summary Statement:**

Quantitative analysis of multicellular development reveals previously hidden functions of an orphan histidine kinase, highlighting the importance of robust phenotyping approaches for understanding bacterial signaling networks.

## Full Text Availability

The license terms selected by the author(s) for this preprint version do not permit archiving in PMC. The full text is available from the preprint server.

